# Regressão do Supradesnivelamento do Segmento ST como Preditor de Reperfusão no Infarto Agudo do Miocárdio: Uma Incógnita Persistente

**DOI:** 10.36660/abc.20210426

**Published:** 2021-07-15

**Authors:** Frederico V. B. Macedo, Domingos Sávio G. Ferreira, Marcelo Augusto A. Nogueira, Victor Raggazzi H. da Silva, Bruno R. Nascimento

**Affiliations:** 1 Faculdade de MedicinaUniversidade Federal de Minas GeraisBelo HorizonteMGBrasilFaculdade de Medicina, Universidade Federal de Minas Gerais, Belo Horizonte, MG - Brasil; 2 Hospital das ClínicasUniversidade Federal de Minas GeraisBelo HorizonteMGBrasilHospital das Clínicas, Universidade Federal de Minas Gerais, Belo Horizonte, MG - Brasil

**Keywords:** Regressão, Supradesnivelamento, Preditor, Reperfusão

A doença isquêmica do miocárdio é a principal causa de morte no mundo, assim como no Brasil.^1^ Sua apresentação de maior gravidade é o infarto agudo do miocárdio com supradesnivelamento do segmento ST (IAMCSST),^2^ que tem na reperfusão precoce a principal terapia para diminuição da mortalidade.^3^ Embora a intervenção coronária percutânea (ICP) primária seja considerada o tratamento “padrão ouro” para o IAMCSST, ela não está suficientemente disponível, e o acesso a ela é ainda desigual.^3,4^ O eletrocardiograma (ECG) é a principal ferramenta para o diagnóstico precoce do IAM e para a decisão sobre a terapêutica a ser implementada.^5^ Considerando-se a facilidade de obtenção, disponibilidade e praticidade do ECG na emergência, a redução do supradesnivelamento do segmento-ST (RST) é proposta como a melhor ferramenta para predizer o sucesso terapêutico pós-trombólise.^6^ No entanto, o método tem reconhecidas limitações, que têm motivado investigações adicionais.

Neste contexto, o artigo “Acurácia da Redução do Segmento-ST Pós-Trombólise como Preditor de Reperfusão Adequada em Estratégia Fármaco-Invasiva”^7^ propõe avaliar as alterações no segmento ST pós-fibrinólise e seu poder de predizer reperfusão adequada utilizando-se os escores angiográficos TIMI-*flow* e o *blush* miocárdico (MBG) como critérios de reperfusão ideal. Nesse estudo, 2.215 pacientes com diagnóstico de IAMCSST foram submetidos à fibrinólise e encaminhados para angiografia em até 24h, ou imediatamente encaminhados à ICP de resgate em caso de insucesso. O ECG foi realizado pré-TNK e 60 min após, e os pacientes foram categorizados em: aqueles com reperfusão ideal (TIMI-3 e MBG-3) e aqueles com reperfusão inadequada (fluxo TIMI <3). Foi definido como critério de reperfusão ao ECG a RST >50%. O critério de reperfusão pelo ECG apresentou valor preditivo positivo (VPP) de 56%; valor preditivo negativo (VPN) de 66%; sensibilidade de 79%; e especificidade de 40%. Houve fraca correlação positiva entre a RST e dados angiográficos de reperfusão ideal (r=0,21; p<0,001) e baixa precisão diagnóstica, com área sob a curva ROC de 0,60 (IC95%; 0,57-0,62). Os resultados evidenciaram que a RST, não conseguiu identificar com precisão os pacientes com reperfusão angiográfica apropriada. Portanto, propõe-se que mesmo pacientes com reperfusão aparentemente bem-sucedida ao ECG devem ser encaminhados à angiografia, para garantir fluxo macro/microvascular adequado.

Em revisão com dados preliminares, Lemos e Braunwald^8^ avaliaram a RST como ferramenta para medir a eficácia da terapia de reperfusão. Observou-se que, apesar de diferenças entre os estudos no que diz respeito às medicações e valores considerados para a RST, esse seria preditor altamente preciso da patência da artéria do infarto (VPP=90%), mas às custas de um VPN de apenas aproximadamente 50%. Lemos e Braunwald^8^ pontuaram ainda que a RST, associada a dor e a concentração sérica de marcadores de necrose miocárdica poderia ser usada para predizer falha na reperfusão, tendo sido propostos 3 critérios: RST< 50% em 90 min, dor torácica persistente em 90 min e relação de mioglobina sérica aos 60 minutos/basal< 4. Entretanto, ainda que a utilização destes critérios em conjunto melhorasse a acurácia, havia um considerável índice de falsos-positivos na predição de sucesso da reperfusão.^8^

Em outro estudo, avaliou-se a RST após trombólise em pacientes com idade maior ou menor que 65 anos com IAMCSST, com a realização de ECGs seriados no seguimento. Nos dias 1 e 2 após o tratamento, o percentual de RST foi maior entre pacientes mais jovens. Além disso, a fração de ejeção ventricular esquerda foi significativamente menor nos mais idosos. Portanto, os dados demonstraram uma pior resposta à terapia trombolítica em pacientes com idade mais avançada, sugerindo que a função sistólica prejudicada nesse grupo pudesse estar associada ao atraso na RST. Ademais, propôs-se que a angiografia e ICP precoces pudessem ser mais indicadas para essa população.^9^ Ainda, estudos que analisaram fatores clínicos relacionados à RST após ICP primária também verificaram melhor resposta em indivíduos mais jovens. Isso nos faz pensar sobre a possibilidade de idosos já terem disfunções microvasculares anteriores ao IAM, contribuindo para a RST incompleta.^10^

No entanto, ainda se busca parâmetros eletrocardiográficos adicionais para aperfeiçoar a acurácia da predição do sucesso terapêutico pós-trombólise. Dotta et al.^11^ analisaram o desempenho da dispersão do intervalo QT como marcador precoce de reperfusão, em adição aos critérios clássicos. A angiografia foi realizada em todos os pacientes, com avaliação do fluxo pelo critério TIMI e MBG da artéria culpada. Os dados sugerem que a dispersão regional do intervalo QT, corrigido pela frequência cardíaca, possa ser um parâmetro útil na identificação não-invasiva de reperfusão.^11^

Dito isso, a revisão da literatura reforça as conclusões do presente estudo.^7^ A RST pós-trombólise isoladamente não define com exatidão os pacientes com reperfusão adequada (Tabela 1). Há alguns fatores clínicos relacionados a menores índices de RST – como a idade avançada e maior tempo de evolução do IAM – que podem indicar realização de angiografia de forma precoce, e novos parâmetros do ECG podem somar ao poder preditivo da RST. Portanto, investigações adicionais são necessárias para se identificar os marcadores ideais de reperfusão angiográfica adequada.

**Tabela 1:** – Sumário de estudos que avaliaram a resolução do segmento ST como critério de sucesso para reperfusão no infarto agudo do miocárdio

Artigos	Principais achados
Acurácia da Redução do Segmento-ST Pós-trombólise como Preditor de Reperfusão Adequada em Estratégia Fármaco-Invasiva^7^	Critério de reperfusão do ECG: redução do segmento ST >50%; VPP de 56%; VPN de 66%; Sensibilidade de 79%; Especificidade de 40%; Conclusão: houve fraca correlação positiva entre a redução do segmento-ST e os dados angiográficos de reperfusão ideal, além de uma baixa precisão diagnóstica (AUC=0,60).
ST segment resolution as a tool for assessing the efficacy of reperfusion therapy^8^	A resolução do segmento ST é um preditor altamente preciso da patência da artéria do infarto (VPP de 90%); No entanto, é impreciso para prever manutenção da oclusão da artéria relacionada ao infarto (VPP de 50%). A associação dessa variável com dor e a concentração sérica de mioglobina podem ser usadas em conjunto para predizer falha na reperfusão. Entretanto, há ainda a limitação dos diagnósticos falsos-positivos de reperfusão.
Resolution of ST-segment Elevation After Thrombolytic Therapy in Elderly Patients With Acute Myocardial Infarction^9^	Idosos parecem ter pior resposta à terapia trombolítica em termos de resolução do segmento ST, o que pode estar associado a uma pior função ventricular esquerda neste grupo; A intervenção coronária percutânea precoce pode ser um método mais aconselhado para essa população.
A Dispersão do Intervalo QT Regional como Preditor Precoce de Reperfusão em Pacientes com Infarto Agudo do Miocárdio Pós-terapia Fibrinolítica ^11^	A dispersão do intervalo QT regional, corrigido pela frequência cardíaca, pode ser um parâmetro do adicional do ECG útil na identificação não invasiva de reperfusão na artéria coronária culpada, 60 min pós-trombólise.

*VPN: valor preditivo negativo; VPP: valor preditivo positivo.*
