# Supplementary material for: Regressão do Supradesnivelamento do Segmento ST como Preditor de Reperfusão no Infarto Agudo do Miocárdio: Uma Incógnita Persistente
Source: Arq Bras Cardiol. 2021 Jul 15;117(1):26–7. [Article in Portuguese] doi: 10.36660/abc.20210426 (PMC8294723; doi:10.36660/abc.20210426)
Supplement: Translation

# Translation: ST-Segment Elevation Regression as a Predictor of Reperfusion in Acute Myocardial Infarction: A Persistent Unknown

**Authors:** Frederico V. B. Macedo, Domingos Sávio G. Ferreira, Marcelo Augusto A. Nogueira, Victor Raggazzi H. da Silva, Bruno R. Nascimento

**Affiliations:** 1 BrasilFaculdade de Medicina, Universidade Federal de Minas Gerais, Belo Horizonte, MG - Brasil; 2 BrasilHospital das Clínicas, Universidade Federal de Minas Gerais, Belo Horizonte, MG - Brasil

**Keywords:** Regression, ST-segment elevation, Predictor, reperfusion

Ischemic heart disease is the leading cause of death worldwide, as well as in Brazil.^1^ Its most severe presentation is the ST-segment elevation myocardial infarction (STEMI),^2^ for which early reperfusion constitutes the main therapy to decrease mortality.^3^ Although primary percutaneous coronary intervention (PCI) is considered the “gold standard” treatment for STEMI, it is not sufficiently available, and access to PCI is still unequal.^3,4^ The electrocardiogram (ECG) is the main tool for the early diagnosis of AMI and for the decision about the ideal therapy to be implemented.^5^ Considering the ease to obtain the ECG, as well as its availability and feasibility in the emergency department, the ST-segment elevation reduction (STR) is proposed as the best tool to predict therapeutic success after thrombolysis.^6^ However, the method has recognized limitations, which have motivated further investigations.

In this context, the article “Accuracy of Post-thrombolysis ST-segment Reduction as an Adequate Reperfusion Predictor in the Pharmaco-Invasive Approach”^7^ aimed to assess changes in the post-fibrinolysis ST-segment and its power to predict adequate reperfusion using the angiographic scores TIMI-flow and myocardial blush grade (MBG) as criteria for ideal reperfusion. In this study, 2,215 patients diagnosed with STEMI were submitted to fibrinolysis and referred for angiography within 24 hours, or immediately referred to rescue PCI in case of failure. The ECG was performed pre-Tenecteplase (TNK) and 60 min after, and the patients were categorized into: those with ideal reperfusion (TIMI-3 and MBG-3) and those with inadequate reperfusion (TIMI flow <3). The reperfusion criteria was defined as an STR >50%. The reperfusion criterion at the ECG showed a positive predictive value (PPV) of 56%; negative predictive value (NPV) of 66%; 79% of sensitivity; and 40% of specificity. There was a weak positive correlation between the STR and the ideal reperfusion angiographic data (r = 0.21; p <0.001) and low diagnostic precision, with an area under the ROC curve of 0.60 (95% CI; 0.57-0.62). The results showed that the STR was unable to accurately identify patients with appropriate angiographic reperfusion. Therefore, it is proposed that even patients with apparently successful reperfusion in the ECG should be referred to angiography, to ensure an adequate macro/microvascular flow.

In a review with preliminary data, Lemos and Braunwald^8^ evaluated the STR as a tool to measure the effectiveness of reperfusion therapy. It was observed that, despite differences between the studies regarding the medications and cutoffsconsidered for the STR, this would be a highly accurate predictor of the patency of the infarcted artery (PPV = 90%), but at the expense of a NPV of only approximately 50%. Lemos and Braunwald^8^ pointed out that the STR, associated with pain and the serum levels of myocardial necrosis markers could be used to predict reperfusion failure, and three criteria were proposed: STR <50% in 90 min, persistent chest pain in 90 min and a ratio of serum myoglobin at 60 minutes/baseline <4. Although the use of these criteria together improved accuracy, there were considerable false-positive rates in predicting reperfusion success.^8^

Another study evaluated the STR after thrombolysis in patients aged 65 years or older with STEMI, with serial ECGs being performed during follow-up. On days 1 and 2 following treatment, the percentage of STR was higher in younger patients. Moreover, the left ventricular ejection fraction was significantly lower in the elderly. Therefore, these data demonstrated a worse response to thrombolytic therapy in older patients, suggesting that impaired systolic function in this group could be associated with delayed STR. Moreover, it was proposed that early angiography and PCI could be more adequate for this population.^9^ Furthermore, studies that analyzed clinical factors related to STR after primary PCI also found a better response in younger individuals. This makes us think about the possibility that elderly people presented microvascular dysfunctions prior to the AMI, contributing to incomplete STR.^10^

However, additional electrocardiographic parameters are still being investigated, to improve the accuracy of post-thrombolysis therapeutic success prediction. Dotta et al.^11^ analyzed the performance of QT-interval dispersion as an early reperfusion marker, in addition to the classic criteria. The angiography was performed in all patients, with flow assessment using the TIMI and MBG criteria of the culprit artery. Data suggest that the regional dispersion of the QT interval, corrected by heart rate, may be a useful parameter for non-invasive identification of reperfusion.^11^

That being said, the literature review reinforces the conclusions of the present study.^7^ Post-thrombolysis STR alone does not accurately stratify patients with adequate reperfusion (Table 1). There are some clinical factors related to lower rates of STR – such as older age and longer duration of AMI evolution – which may indicate early angiography, and new ECG parameters may add to the predictive power of STR. Therefore, further investigations are necessary to identify the ideal markers of adequate angiographic reperfusion.

**Table 1:** – Summary of studies that evaluated ST-segment resolution as a success criterion for reperfusion in acute myocardial infarction

Articles	Main findings
Acurácia da Redução do Segmento-ST Pós-trombólise como Preditor de Reperfusão Adequada em Estratégia Fármaco-Invasiva^7^	ECG reperfusion criteria: ST-segment reduction >50%; 56% PPV; 66% NPV; 79% sensitivity; 40% specificity; Conclusion: there was a weak positive correlation between ST-segment reduction and ideal reperfusion by angiographic data, in addition to a low diagnostic accuracy (AUC = 0.60).
ST segment resolution as a tool for assessing the efficacy of reperfusion therapy^8^	ST segment resolution is a highly accurate predictor of infarct artery patency (90% of PPV); However, it is inaccurate to predict persistent occlusion of the infarct-related artery (50% of PPV). The association of this variable with pain and the serum levels of myoglobin can be used together to predict reperfusion failure. However, there is still a limitation of false-positive diagnoses of reperfusion.
Resolution of ST-segment Elevation After Thrombolytic Therapy in Elderly Patients With Acute Myocardial Infarction^9^	Elderly people seem to have a worse response to thrombolytic therapy in terms of ST-segment resolution, which may be associated with worse left ventricular function in this group; Early percutaneous coronary intervention may be a more recommended method for this population.
A Dispersão do Intervalo QT Regional como Preditor Precoce de Reperfusão em Pacientes com Infarto Agudo do Miocárdio Pós-terapia Fibrinolítica ^11^	Dispersion of the regional QT-interval, corrected by heart rate, may be an additional useful ECG parameter for non-invasive identification of reperfusion in the culprit coronary artery, 60 min post-thrombolysis.

*NPV: negative predictive value; PPV: positive predictive value.*
